# Optimization of dual duct forced auxiliary ventilation system to mitigate particulate matter emissions in a polymetallic underground mine environment: A hybrid approach

**DOI:** 10.1371/journal.pone.0322278

**Published:** 2025-05-02

**Authors:** Abdullah Rasheed Qureshi, Sergei Sabanov, Emil Bayramov, Jessica Neafie

**Affiliations:** 1 School of Mining and Geosciences, Nazarbayev University, Astana, Kazakhstan; 2 School of Science and Humanities, Nazarbayev University, Astana, Kazakhstan; NED University of Engineering and Technology, PAKISTAN

## Abstract

The auxiliary ventilation system (AVS) is essential for managing airflow and reducing particulate matter (PM) levels in underground mine environments. Despite its importance, prior studies have insufficiently examined the optimal design of dual duct forced (DDF) AVS to improve airflow and PM management during the loading and unloading operations of diesel-powered equipment (DPE). This work addresses the research gap by utilizing a hybrid methodology to assess the effectiveness of four DDF-AVS designs (1–4) under two distinct DPE operating scenarios: (S1) DPE loading beside the working face and (S2) DPE unloading at a temporary dumpsite. The study utilized Ansys-Fluent for numerical simulations and revealed the following conclusions: the airflow field within the drift displays intricate patterns that substantially affect PM transport; S2 presents the greatest potential PM exposure danger to DPE operators, succeeded by S1. Among the designs, AVS 2 and AVS 3 exhibited efficiency by less complicated airflow patterns and optimal PM transposition within the drift. In comparison to AVS 1, the PM dispersion enhanced by 15.66% and 7.83% in S1, whereas in S2, it improved by 27% and 46% under AVS 2 and AVS 3, respectively. This research study offers significant insights for optimizing AVS designs, minimizing PM exposure to miners, and improving the underground mine environment through cleaner production techniques.

## 1. Introduction

Mining operations involve the extraction and transportation of precious resources for further processing. These activities produce hazardous gases, dust, and PM, which contaminate the inside and surrounding mine environment [1]. The mine transportation system is mainly dependent on DPE, but the emission from the exhaust tailpipe of DPE is one of the major PM sources and poses a significant threat to human health in underground mines [2–4]. Adsorption, absorption, and homogenous nucleation are the primary composing factors of PM. PM has a particle size distribution ranging from 5 nm to 10 µm, and most of these particles fall in the range between 0.1and 1 µm [5–7]. Because of its small size, PM floats in the air for extended periods, and prolonged exposure to PM concentrations can cause respiratory and circulatory system disorders, potentially leading to cancer [8–10].

**Fig 1:**
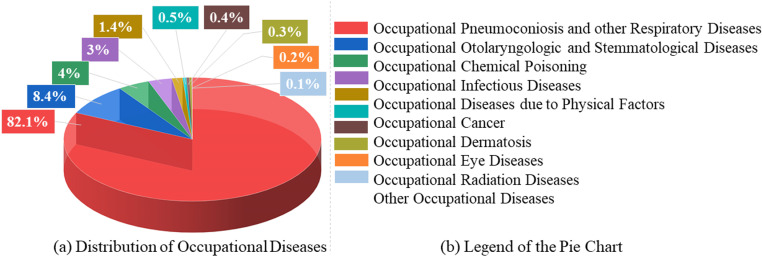
(a) Distribution of Occupational Diseases, and (b) Legend of the Pie Chart.

A recent study by the Harvard T. H. Chan School of Public Health established a cause-and-effect connection between prolonged exposure to fine PM and untimely mortality [11]. The National Institute for Occupational Safety and Health (NIOSH), USA, categorizes PM as an occupational carcinogen, and the International Agency for Research on Cancer (IARC) defines PM as a class I human carcinogen [12,13]. As a result, the Occupational Safety and Health Administration (OSHA) recommends a 10 mg/m^3^ occupational exposure limit for PM during an 8-hour time-weighted average (TWA) in the air of a workplace [14,15]. Moreover, Fig 1, modified from [16], shows the overall distribution of occupational disease cases, and more than 80% of cases were reported for “Pneumoconiosis and other Respiratory Diseases.”

Additionally, PM exposure poses greater health risks in underground mines due to limited airflow and confined spaces that hinder elimination of PM and other aerosols. Therefore, it is of utmost importance to investigate PM mitigation characteristics in underground mines.

Underground mines generally use the AVS to control PM in roadways, working faces, and near dead ends [17]. Recent research created numerous operating scenarios to evaluate the PM emissions from the exhaust tailpipe of DPE under a single-duct forced (SDF) AVS or a dual-duct hybrid (DDH) AVS in underground mines. However, researchers usually adopted a combination of field experimentation and numerical simulation to overcome the challenges associated with experimental approaches while quantifying and mitigating the PM in underground mines.

For instance, Duan et al. investigated the diffusion and distribution characteristics of PM emitted from the exhaust tailpipe of DPE in a heading face of an underground coal mine. An SDF-AVS was installed to supply required airflow to the heading face. The results revealed that the PM mainly accumulated in the blind zone of airflow, and the distribution characteristics were maximum in the middle and minimum on both sides of the single-end roadway [18]. Chang et al. investigated PM dispersion characteristics emitted from the exhaust tailpipe of DPE during shotcreting, charging, and loading activity in an underground development face. The development face was equipped with SDF-AVS. The results revealed regions with higher PM concentrations, the efficiency of the existing SDF-AVS on PM dilution, and a proposal to increase the volumetric airflow during loading activity in the development face [19]. Nie et al. studied the PM diffusion and distribution emitted from DPE under different driving speeds of DPE in an underground coal mine roadway. The results show the optimized diffusion distance between the DPE velocity and PM concentration. The diffusion distance less than 3 m was most critical, followed by the distance between 5 and 15 m in the roadway [20]. Chang et al. conducted a study on the working face of an underground mine in Western Australia. The working face was equipped with SDF-AVS, and the impact of AVS was investigated on the distribution of PM concentration and its diluting effect. Additionally, an adjustment was proposed between the distance of AVS’s outlet and the working face to achieve the optimum dilution rate [21,22].

Zhou et al. investigate PM and tail gas distribution under the influence of DDH-AVS in a fully mechanized working face. The study also examined the impact of air suction volume and distance between DPE and the heading face on the diffusion law of PM and exhaust gas. The results showed that the closer the distance between the DPE and the heading face, the higher the volumetric airflow required to dilute the hazardous pollutants. In contrast, the lower volumetric airflow is sufficient to dilute the PM and tail gas if the distance between the heading face and DPE increases [23]. In another study, Zhou et al. analyzed the pollution law for dust from the heading face and PM from the exhaust tailpipe of DPE under DDH-AVS in a mechanized excavation face. A few operating scenarios were created based on the air intake volumes of the dust removal (suction) fan and the distance between the outlet of the dust removal fan and the heading face by adopting CFD. The results revealed that the best control effects can be achieved on dust and PM under different volumetric air intakes when the distance between the outlet of the dust removal fan and heading face is 5 m [24].

In the above studies, researchers commonly focused on underground coal mines and used SDF-AVS or DDH-AVS to determine the optimal airflow rate and the distance of the air duct from the excavation face to control PM near the mechanized working face. However, SDF or DDH-AVS may not be effective in efficiently ventilating a working face cut by drill and blast because the AVS outlet should be installed at a considerable distance to avoid any damage to the AVS. Moreover, these AVS may also not be adequate in ventilating a temporary dumpsite or an idling chamber in a drift of an underground mine. Thus, this study explores the airflow migration and PM transposition near the working face as well as inside the temporary dumpsite or idling chamber in a drift, under the influence of DDF-AVS.

To maintain a safe PM concentration during the loading and unloading of blasted ore, it is necessary to have a very effective and substantial ventilation system. However, employing the SDF-AVS necessitates the installation of powerful fans, which will generate a high-velocity airflow that surpasses the permissible wind speed at the outlet of the duct. Moreover, these high-speed, powerful jet fans may also increase the residence time of ultra-fine particles near the working face due to recirculation. Therefore, the DDF-AVS was suggested. Currently, the DDF-AVS has been implemented in the field. However, important characteristics such as the airflow field and the concentration distribution of PM near the dead-ends during the loading and dumping of the blasted ore have not yet been established. Furthermore, when it comes to on-site applications, the airflow ratio and the location of the air pipe are often determined using experiential knowledge. To effectively implement this ventilation approach, it is crucial to carry out a thorough study on the ventilation properties and ideal parameters of the DDF-AVS in the underground mine.

## 2. Materials and methods

### 2.1. Research framework

**Fig 2:**
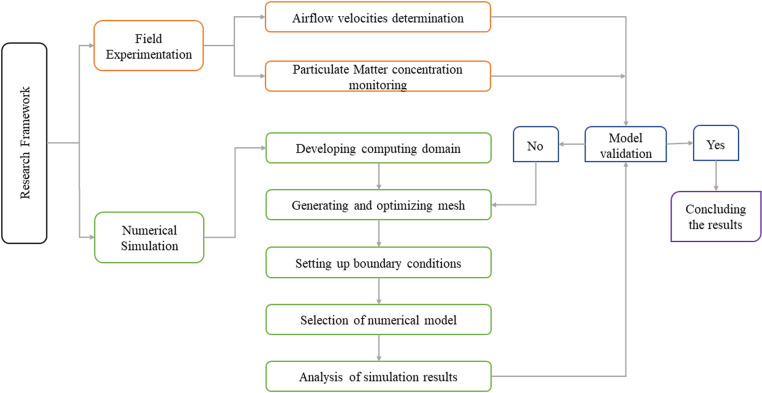
Research framework overview.

The research framework adopted in this research study is shown in Fig 2. The field experimentation was conducted while the DPE was loading the ore near the working face (S1) and unloading the ore inside the temporary dumpsite (S2). A hand-held anemometer (Alnor Rotating Vane RVA501) was used to calculate the airflow velocities. DustTrak DRX Aerosol Monitor 8533 was adopted to record the real-time PM concentrations. Afterwards, an Ansys-Fluent solver was selected to conduct the numerical simulations. A computational geometry domain of the mine drift was constructed based on the dimensions measured during the field visit. Subsequently, the distribution of the computing domain in small finite volume cells (mesh) was carried out and the boundary conditions were assigned. The Eulerian-Lagrangian model was selected to simulate the airflow velocities and particle trajectories in the computational domain. Following to that, the numerical simulation model was validated by comparing the results with field experiment. After achieving the validation criteria, the numerical simulation was evaluated, and predicted results were used to analyze the impact of DDF-AVS on the diffusion characteristics of PM under the actual airflow velocity. Moreover, four DDF-AVS positions were evaluated in the simulation to assess their effectiveness in reducing potential PM exposure.

### 2.2. Mine site study area

In this study, the airflow velocities and real-time PM sampling were carried out in an underground mine located in the Eastern Kazakhstan region. The capacity of the underground polymetallic mine was 300 thousand tons of ore per year.

#### 2.2.1. Data monitoring equipment.

This study employed an anemometer to measure airflow velocity at designated points throughout time for airflow analysis. Furthermore, for PM analysis, the “DustTrak DRX Aerosol Monitor 8533” was employed to quantify both mass and size distributions of aerosols under PM conditions. The DustTrak is a 90° light scattering device that quantifies particulate size fractions (PM1, PM2.5, PM4, PM10) and mass concentration, facilitating real-time aerosol surveillance. The mass concentration fluctuates between 0.001 and 150 mg/m3 with an accuracy of ±5%, whilst the particle size spans from 0.1 to 15 µm [25–27]. 2.3 Computing domain and geometric model description.

#### 2.2.2. Construction of geometric model.

**Fig 3:**
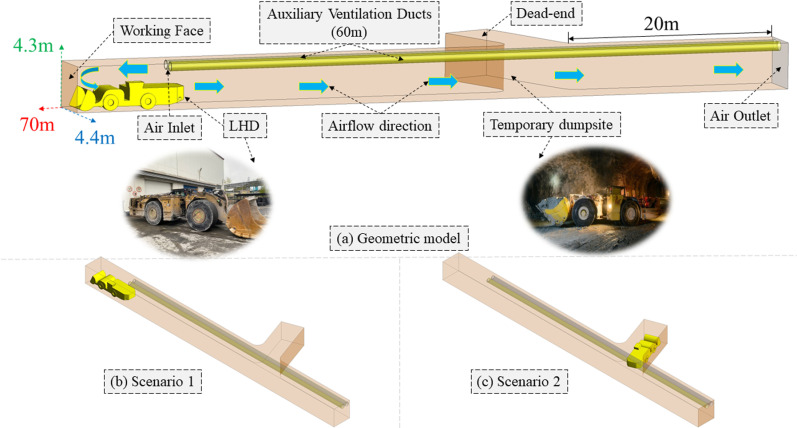
(a) Geometric model of the mine drift, (b) S 1, and (c) S 2.

This study adopted Geometry in Space-Claim of Ansys-Fluent (2020 R2) to conduct equal scale modelling on the drift of the underground polymetallic mine. In addition, the model was appropriately simplified based on calculation time, convergence, and computational complexity. As illustrated in Fig 3 (a), the geometric model dimensions were 70 m length in total, 4.4 m width and 4.3 m height, respectively. The temporary dumpsite was 20 m from the air outlet. Further, it contained DDF-AVS extended up to 60 m into the drift from the entrance and the outlet of both ventilation ducts was 10 m away from the active working face. A load-haul-dump (LHD) Caterpillar R-1700 (10 m length, 2.9 m width, and 2.5 m height) performed the loading and unloading operations in the drift. Fig 3 (b and c) shows the S1 and S2, respectively.

#### 2.2.3. Evaluation of DDF-AVS designs.

**Fig 4:**
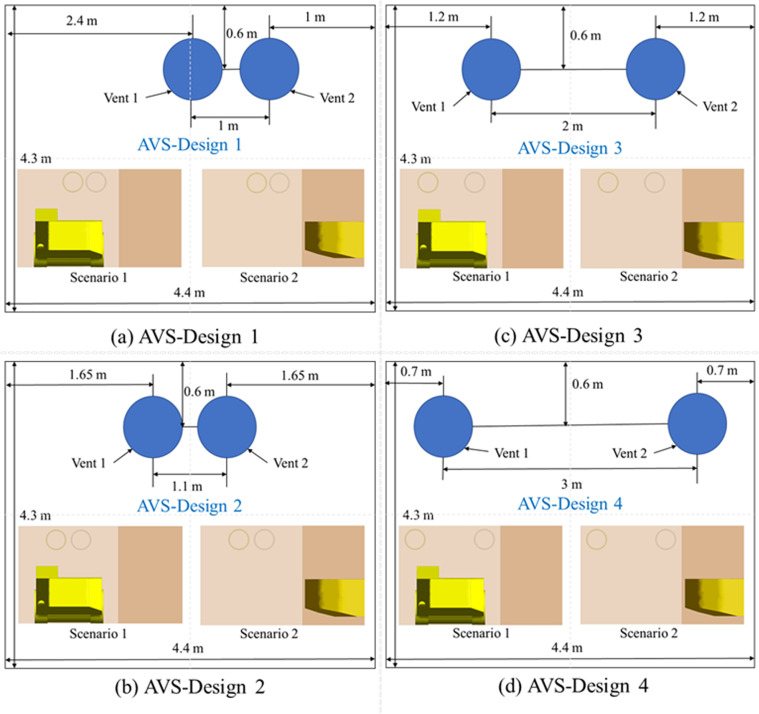
(a) AVS 1, (b) AVS 2, (c) AVS 3, and (d) AVS 4 for both scenarios.

The distance between the central axes of the AVS’s outlets and the location of the outlets in relation to the central axis of the mine drift along the z-axis are two design factors included in the DDF-AVS system. Fig 4 depicts several different design configurations that were developed to evaluate the influence of these parameters on the airflow field and the transposition of PM to optimize the DDF-AVS.

In AVS 1, Fig 4 (a), the outlets are separated by 1 m, with Vent 1 situated 2.4 m from the left wall and Vent 2 situated 1 m from the right wall. An illustration of AVS 2 (Fig 4 (b)), shows that the outlets are spaced 1.1 m apart, and both vents are located 1.65 meters from the respective right and left walls. Then in AVS 3, Fig 4 (c), the outlets are separated by a distance of 2 m, and both vents are positioned 1.2 m away from the side walls. Lastly, the AVS 4in Fig 4 (d), the outlets are separated by a distance of 3 m, and each vent is located 0.7 m away from the side walls.

### 2.4. Mathematical approach

Generally, there are two models in CFD, which are commonly adopted to simulate the two-phase flow [28]. The first model is known as Species Transport, which is based on Eulerian-Eulerian (fluid-fluid) approach. In this model, the PM and the airflow are both considered as a continuous phase [29,30]. The second model is the Discrete Phase Model (DPM), which adopts the Eulerian-Lagrangian approach (particle-fluid) and treats PM as a solid particle and airflow as a fluid flow [31–33]. A few of the simulation-based studies conducted to compare the results between both the CFD continuous phase model and DPM. The authors concluded that both models are feasible to simulate a solid-gas scenario. However, the DPM has better agreement with experimental results compared to the continuous model [34–36]. As far as PM is concerned, numerical research studies were conducted and the authors used both continuous and discrete phase models to compare the diesel PM distribution in the underground mine. Similarly, they claimed that a better agreement was drawn between the experimental results and, rather than continuous phase model. However, the computational cost related to continuous phase is comparatively less than the DPM [21,37] because the Species Transport Model deals with continuous fields. On the contrary, the DPM involves particle motion tracking and interaction with fluid and other particles. Thus, in this research study the Eulerian-Lagrangian approach was used to simulate the airflow and PM in the underground mine.

#### 2.4.1. Governing equations.

##### 2.4.1.1. Airflow mathematical model

The airflow was treated as a continuous phase and incompressible fluid. The equations for mass conservation [38,39] and momentum [40,41] can be written as equation 1 and 2:


$$\frac{\partial \rho }{\partial t}+\nabla .\left(\rho \vec{v}\right)=S_{m}$$ (1)

$$\frac{\partial }{\partial t}\left(\rho \vec{v}\right)+\nabla .\left(\rho \vec{v}.\vec{v}\right)=\;-\nabla p+\nabla .\left(\overset{―}{\overset{―}{\tau }}\right)+\rho \vec{g}+\vec{F}$$ (2)

Where, p, $\overset{―}{\overset{―}{\tau }}$, $\rho \vec{g}$, $\vec{v},$ S_m_, $\vec{F}$ are the static pressure, stress tensor, gravitational body force, velocity vector, added mass form dispersed phase to the continuous phase and external body forces, respectively.


$$\overset{―}{\overset{―}{\tau }}=\mu \left[\left(\nabla \vec{v}+\nabla {\vec{v}}^{T}\right)-\frac{2}{3}\nabla \vec{v}I\right]$$ (3)

Where, μ and I are molecular viscosity, and unit tensor.

The standard model of turbulent kinetic energy (k) and dissipation rate (ε) are usually considered to simulate the turbulent flow [41–44].

The k-equation:


$$\frac{\partial (\rho k)}{\partial t}+\frac{\partial (\rho ku_{i})}{\partial x_{i}}=\frac{\partial }{{\partial x}_{j}}\left[\left[\mu +\frac{\mu_{t}}{\sigma_{k}}\right]\frac{\partial k}{{\partial x}_{j}}\right]+G_{k}-\rho \varepsilon$$ (4)

The turbulent viscosity $\mu_{t}$ can be determined as follows:


$$\mu_{t}=\rho C_{\mu }\frac{k^{2}}{\varepsilon }$$ (5)

The ε equation:


$$\frac{\partial (\rho \varepsilon )}{\partial t}+\frac{\partial (\rho \varepsilon u_{i})}{\partial x_{i}}=\frac{\partial }{{\partial x}_{j}}\left[\left[\mu +\frac{\mu_{t}}{\sigma_{\varepsilon }}\right]\frac{\partial \varepsilon }{{\partial x}_{j}}\right]+\frac{C_{1\varepsilon }}{k}-C_{2\varepsilon }\rho \frac{\varepsilon^{2}}{k}$$ (6)

Where, ρ, k, ε, $\mu ,\;\mu_{t},$ $G_{k},$ are the density, turbulent kinetic energy, turbulent energy dissipation rate, laminar viscosity coefficient, turbulence viscosity coefficient, and average velocity gradient, respectively. The constant values of terms $C_{1\varepsilon },C_{2\varepsilon },C_{\mu },\;\sigma_{k},\sigma_{\varepsilon }$ are 1.44, 1.92, 0.09, 1.00, and 1.30, respectively.

##### 2.4.1.2. Particle motion mathematical model

ANSYS Fluent discrete phase model (Lagrangian) predicts the trajectory of particles by integrating the balance force on the particle. This force equates the particle inertia with the forces acting on the particle and can be written as [41,24]:


$$m_{p}\frac{{d\vec{u}}_{p}}{dt}=m_{p}\frac{{\vec{u}-\vec{u}}_{p}}{\tau_{r}}+\;m_{p}\frac{\vec{g}\;\left(\rho_{p}-\rho \right)}{\rho_{p}}+\vec{F}$$ (7)

where $m_{p}$, $\vec{u}$, ${\vec{u}}_{p}$, ρ, $\rho_{p}$, $\vec{F}$, $m_{p}\frac{{\vec{u}-\vec{u}}_{p}}{\tau_{r}}$, and $\tau_{r}$ are the particle mass, fluid phase velocity, particle velocity, fluid density, density of the particle, an additional force, drag force, and droplet or particle relaxation time, respectively. The $\tau_{r}$ can be calculated by:


$$\tau_{r}=\frac{\rho_{p}d_{p}^{2}}{18\mu }\frac{24}{C_{d}Re}$$ (8)

Here, μ, $d_{p}$, and Re are molecular viscosity of the fluid, particle diameter, and relative Reynolds number, which can be numerically defined as:


$$Re\;=\;\frac{{\rho d}_{p}\;\left|{\vec{u}}_{p}-\vec{u}\right|}{\mu }$$ (9)

### 2.5. Mesh division and independence assessment

**Fig 5:**
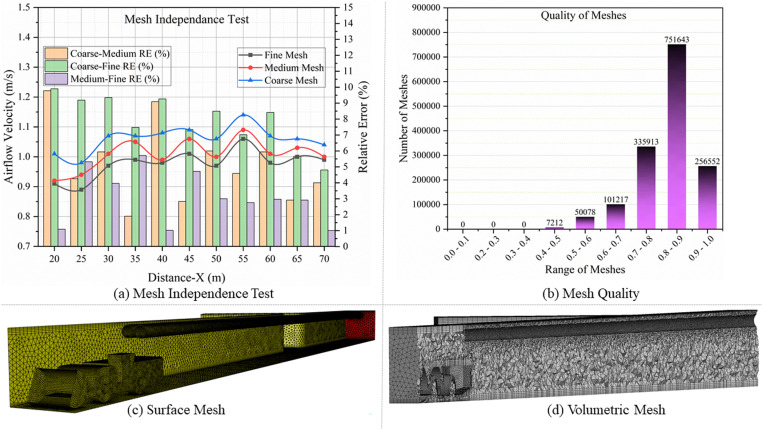
(a) Mesh independence test, (b) Mesh quality, (c) Surface mesh, and (d) Volumetric mesh.

An essential factor influencing the simulation outcomes is the quality of the mesh. In general, a higher mesh density in a simulation leads to more precise findings. However, the duration and cost of computation will increase. Hence, this work consisted of three sets of tetrahedral meshes with varying numbers, all of which were configured to have an average skewness of 0.3. These three categories of meshes were designated as Fine Mesh, Medium Mesh, and Coarse Mesh. The mesh sensitivity analysis conducted while keeping the mesh ratio not less than 1.26 [45]. The relevant mesh quantities of the Fine Mesh, Medium Mesh, and Coarse Mesh were set as 1922954, 1502615, and 1186915, respectively. Additionally, the height of the initial mesh cell was established at 0.02 m, based on the calculation of dimensionless y+ =30 [46]. The total number of prism layers was established at 5, and the inflation ratio of each layer was set at 1.2. Ansys-Fluent was utilized to model the airflow in the drift with various mesh configurations. Using CFD-Post, the line monitors were used to calculate the airflow velocity. The line monitors were drawn on x, y, and z coordinates in the following order: L1(x, 1.5, 3.4), L2(x, 1.5, 2.2), L3(x, 1.5, 1), L4(x, 2.2, 1), L5(x, 2.2, 2.2), and L6(x, 2.2, 3.4). Fig 5 (a) shows the results of the mesh independence test, with the blue, red, and black lines representing the airflow velocity in Coarse, Medium, and Fine Meshes, respectively. The brown, green, and purple bars illustrate the relative error [47] between Coarse and Medium Meshes, Coarse and Fine Meshes, and Medium and Fine Meshes, respectively. Fig 5(b) depicts the overall number of meshes and the relevant quality of the meshes, and Fig 5(c and d) shows the appearance of surface and volumetric meshes (Medium Mesh) of the computational domain.

The analysis shows that the fluctuation pattern of the airflow velocity acquired from the three meshes is nearly uniform. The simulation results from the Fine Mesh and Medium Mesh exhibit a consistent fluctuation trend, and the data they provide are nearly identical. The maximum relative error between Fine Mesh and Medium Mesh was less than 6%. However, the Coarse Mesh exhibits a significant divergence, and the maximum relative error between Fine Mesh and Coarse Mesh is 10%, and between the Medium Mesh and Coarse Mesh is 9.5%. Therefore, to achieve more precise simulation findings and save computational expenses in the study, Medium Mesh is used for the numerical simulations.

### 2.6. Parameter settings and boundary conditions

To study airflow migration and PM diffusion behaviors in an underground mine, it is necessary to establish suitable fundamental parameters and various boundary conditions. Table 1 provides a comprehensive list of the fundamental parameters and boundary conditions employed in this simulation. This study adopted a steady state simulation and the supplied volumetric airflow was considered incompressible, and the heat transfer between the objects was not considered. The outlets of DDF-AVS were considered “velocity inlets” and the exit of the mine drift was selected as the “pressure outlet”.

**Table 1:** Boundary condition and parameter settings.

Boundary condition	Parameter settings	Boundary condition	Parameter settings
Model	k-epsilon	PM DPE exhaust flow rate	3.11e-6 (kg/s)
Near-wall treatment	Standard wall function	Maximum diameter	1e-6 (m)
Velocity inlet 1 and 2	17 m/s	Minimum diameter	1e-8 (m)
Pressure outlet	101.325 kPa	Mean diameter	4.9e-7 (m)
Discrete Phase	On	Solution scheme	Coupled
Diameter Distribution	Rosin-Rammler	Momentum	Second order upwind
Stochastic tracking	Discrete random walk	Turbulent kinetic energy	Second order upwind
PM mucking flow rate	6.75e-5 (kg/s)	Turbulent dissipation rate	Second order upwind
PM mucking source (x,y,z) coordinates	(1, 1.2, 2.2)	PM DPE exhaust source (x,y,z) coordinates	(10.5, 0.9, 3.4)

## 3. Results and analysis of airflow and PM simulation

The CFD-Post program facilitated the visualization of airflow and PM simulation data, which were subsequently examined. Multiple outcomes were achieved by using planes, contours, and vectors options.

### 3.1. Airflow field analysis

**Fig 6:**
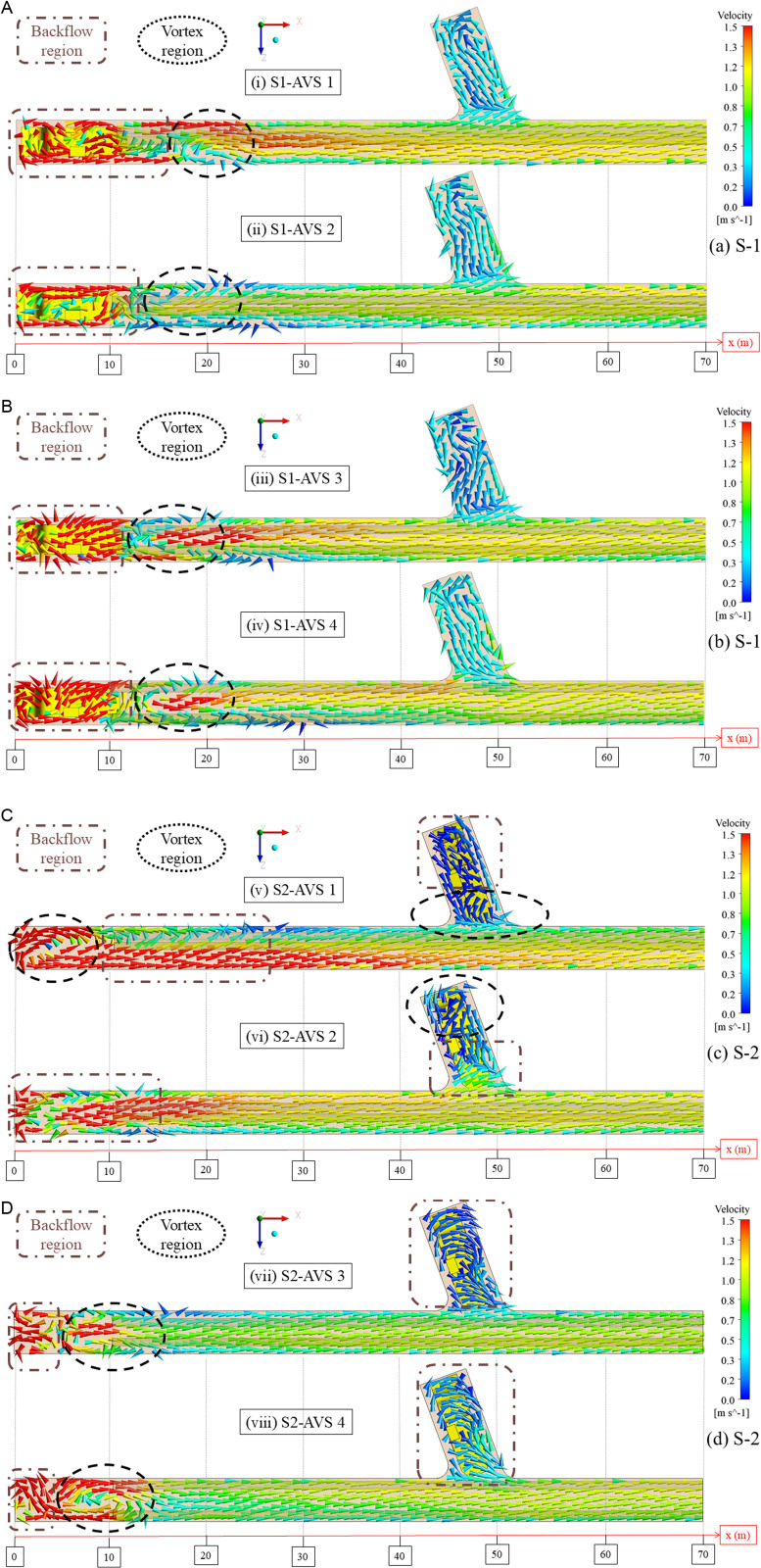
Airflow field analysis in AVS-Designs 1-4 (a and b) S1 and (c and d) S2.

Fig 6 illustrates the airflow field at 2.2 m above the ground, corresponding to the breathing zone of the DPE operator. The DDF-AVS system efficiently directs fresh airflow to the working face. Upon encountering this interface, the airflow reverses, redirecting it toward the exit of the drift, thus establishing a “Backflow” zone. The interaction between airflow and the working face leads to a significant decrease in momentum. Moreover, the presence of the DPE significantly reduces the airflow momentum. The sudden reduction in momentum produces a “Vortex” region that captures a significant volume of airflow and establishes a concentrated zone of negative pressure behind the DPE. These zones are essential in governing the distribution and transportation of PM inside the mine.

1. In (i) S1-AVS 1, the Backflow region extends more than 10 m from the working face, whereas the Vortex region spans between 10 and 30 m. The Vortex region with low airflow momentum is formed near the left-side wall, while the airflow with high momentum passes along the right-side wall. In (v) S2-AVS 1, the absence of DPE lead the formation of a larger Backflow region near the working face that extends more than 20 m, whereas another Backflow region exists deep inside the temporary dumpsite above the DPE. The Vortex region with low airflow momentum exists near the working face and in the center of the drift, covering less than 10 m, and another Vortex region exists at the entrance of the temporary dumpsite, the back of the DPE. The dynamic positions of the DPE account for the positions and dimensions of these regions. Moreover, the airflow patterns partially stabilize between 30 and 70 m of the drift.
2. In (ii) S1-AVS 2, the Backflow region continues up to 10 m, whilst a Vortex region is observed between 10 and 20 m. In the Vortex region, a lower airflow momentum exists near both the side-walls, while leaving a higher airflow momentum in the center of the drift. Between 20 and 70 m within the drift, the airflow exhibits an approximately stable trend. In (vi) S2-AVS 2, the Backflow region adjacent to the working face exceeds 10 m, and an additional Backflow region is observable near the entrance of the temporary dumpsite, behind the DPE. The Vortex region is notably lacking at the working face due to the lack of the DPE and the peculiarities of the DDF-AVS design; However, a Vortex region forms deep inside the temporary dumpsite, in front of the DPE. Beyond the Backflow region, the airflow in the drift exhibits partially stable characteristics.
3. In (iii) S1-AVS 3, the Backflow region extends to approximately 10 m, whereas the Vortex region is identified between 10 and 20 m. The Vortex region displays differing airflow magnitudes, characterized by low momentum adjacent to the right-side wall, medium momentum near the left-side wall, and elevated airflow magnitude at the center of the drift. In (vii) S2-AVS 3, the Backflow region is limited to under 10 m, whereas a second Backflow region extends across nearly the whole temporary dumpsite of the drift. The Vortex region is located between 10 and 20 m, characterized by low airflow magnitude adjacent to the left-side wall. Furthermore, the drift exhibits a consistently stable airflow pattern from 20 to 70 m.
4. In (iv) S1-AVS 4, the Backflow region generally extends approximately 10 m, whereas the Vortex region ranges from 10 to 20 m. In the Vortex region, comparable airflow magnitudes are noted along both sidewalls, leading to an increased airflow amplitude at the center of the drift. In S2-AVS 4, the Backflow region restricted to under 10 m near the working face, while the secondary Backflow region that spans almost the entire temporary dumpsite of the drift. The Vortex region is located between 10 and 20 m, exhibiting reduced airflow magnitude adjacent to the left-side wall and increased magnitude near the right-side wall. The airflow demonstrates a partially stable behavior between the 20 and 70 m of the drift.

The evaluation of the airflow field shows that the type of DDF-AVS designs and the presence of the DPE significantly modify airflow momentum and characteristics. Among the AVS designs, AVS 2 exhibits enhanced performance by reducing the Vortex zone and facilitating stable airflow, whereas other variants display differing levels of turbulence and flow instability. These findings highlight the essential importance of AVS configuration and DPE positioning in enhancing airflow patterns and reducing PM concentration.

### 3.2. Airflow velocity distribution in x, y, and z coordinates

**Fig 7:**
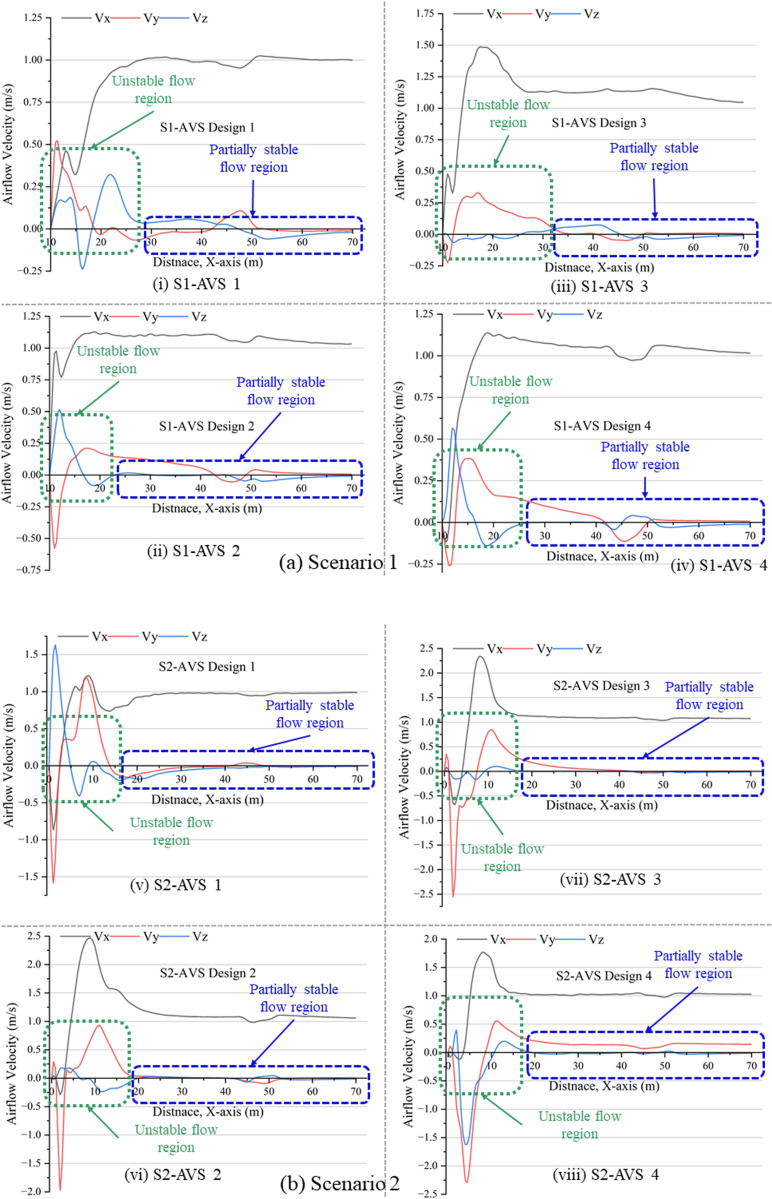
Airflow velocity (Vx, Vy, Vz) in X, Y, and Z dimensions on line monitors (a) S1 and (b) S2.

The airflow in the drift is turbulent because of dynamic mining activities and the intricate geometry of the mine drift. As illustrated in Fig 7 (a and b), assessing the airflow velocity in the x, y, and z directions along the six-line monitors would yield insights into the intensity and direction of airflow in the drift, which typically induces the formation of eddies. The line monitors are designated as L1 (x, 1.5, 3.4), L2 (x, 1.5, 2.2), L3 (x, 1.5, 1), L4 (x, 2.2, 1), L5 (x, 2.2, 2.2), and L6 (x, 2.2, 3.4). The average of all six lines in each AVS designs and in both scenarios was utilized to record the airflow velocity in the x-direction (Vx), y-direction (Vy), and z-direction (Vz). In S1, the line monitors ranged from 11 to 70 m due to the DPE being positioned near the working face; however, in S2, the line monitors commenced from 0 to 70 m since the DPE was located within the temporary dumpsite. Based on the Vx, Vy, and Vz components of airflow velocity, the mine drift is categorized into “Unstable flow region” and “Partially stable flow region.”

1. The S1-AVS 1 delineates the unstable flow area between 10 and 30 meters. In this region, Vx approached 1 m/s, Vy attained 0.5 m/s, while Vz varied between -0.25 and 0.4 m/s, respectively. In the zone of partially steady flow, these components exhibit negligible changes. The (v) S2-AVS 1 indicates the unstable flow area between 0 and 25 m. In this region, Vx varied from -0.75 to 1 m/s, Vy varied from -1.5 to 1 m/s, and Vz varied from 1.5 to -0.25 m/s, respectively.
2. The (ii) S1-AVS 2 exhibits an unstable flow zone between 10–22 m, whereas the remainder of the drift is characterized by a somewhat stable flow region. In the unstable flow area, Vx exceeded 1 m/s, whereas Vy varied between -0.5 and 0.25 m/s, and Vz oscillated between 0.5 and -0.1 m/s, respectively. The (vi) S2-AVS 2 demonstrates the unstable flow zone at a distance of less than 20 m. The Vx varies from -0.25 to 2.5 m/s, Vy ranges from -2–1 m/s, and Vz oscillates between 0.25 and -0.25 m/s.
3. The (iii) S1-AVS 3 exhibits an unstable flow zone from 10 to 25 m, whereas the remainder of the drift is characterized by a somewhat stable flow region. In the unstable flow area, Vx exceeded 1.5 m/s, Vy varied between –0.25 and 0.35 m/s, and Vz remained approximately 0 m/s. The (vii) S2-AVS 3 indicates the unstable flow zone ranging from 0 to 15 m. The Vx attained a maximum of 1.75 m/s, the Vy varied between -2.5 and 0.75 m/s, and the Vz remained approximately 0 m/s.
4. The (iv) S1-AVS 4 exhibits an unstable flow area from 10 to 25 m, while the remainder of the drift is in a somewhat stable flow region. In the unstable flow area, Vx exceeded 1.1 m/s, whereas Vy varied between -0.25 and 0.35 m/s, and Vz oscillated between 0.5 and -0.12 m/s, respectively. The (viii) S2-AVS 4 indicates the unstable flow zone is under 20 m. The Vx attained a maximum of 1.75 m/s, whereas Vy varied between -2.12 and 0.5 m/s, and Vz oscillated between 0.3 and -1.5 m/s, respectively.

The findings indicated that the least unstable flow zone is located in AVS 2 and 3 in both cases. The Vx is almost identical with slight variations. The Vy and Vz are more influential in generating turbulent and unstable flow zones. In AVS 2 and 3, the influence of Vz is practically insignificant, resulting in barely any turbulence effects in the Z-direction of airflow. However, the variations in the Vy direction are usually due to the airflow mechanism.

### 3.3. Airflow velocity vectors distribution characteristics on monitoring planes

**Fig 8:**
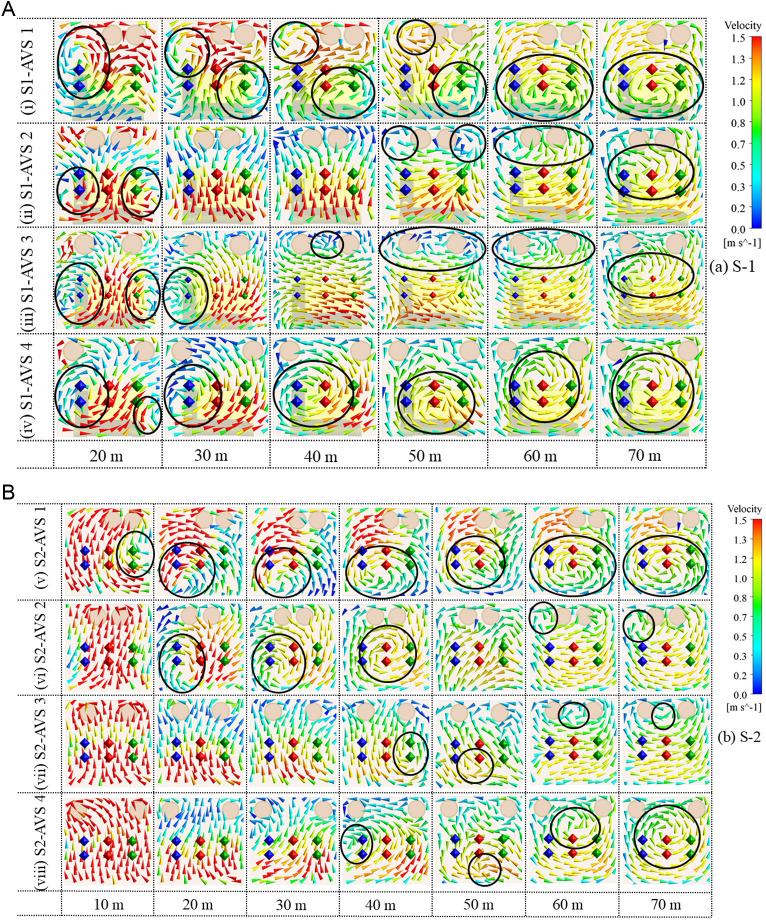
Airflow velocity vectors representation on monitoring planes (a) S1 and (b) S2.

The airflow velocity vectors illustrate the direction of airflow at each monitoring plane in both cases, as depicted in Fig 8 (a, b). These vectors facilitate the comprehension of airflow behavior across all monitoring planes under all the AVS designs. The blue, red, and green dots on the monitoring planes signify the data monitoring points, while the black circles denote the eddies on the monitoring planes.

1. The S1-AVS 1 has a significant eddy formation near the left-side wall at 20 m. It subsequently propagated into two eddies at 30, 40, and 50 m. At 60 and 70 m, two substantial eddies formed, partially covering drift. In (v) S2-AVS 1, no eddy was seen at 10 m due to the lack of the DPE. Furthermore, a minor eddy was established in the lower left-side of the drift. Subsequently, this eddy expanded in size and encompassed nearly center of the drift from 30 to 70 m.
2. The (ii) S1-AVS 2 exhibits two minor eddies located on the left and right-side walls at 20 m. Furthermore, no eddies were present at 30 and 40 m. Subsequently, two minor eddies emerged on the upper left and right sides of the drift at 50 m, which then merged into a single eddy encompassing both AVS at 60 m. The substantial eddy propagated downhill, encompassing the center of the drift at 70 m. The S2-AVS 2 displays no eddy at 10 m. An insignificant eddy formed near the floor at 20 m, which progressively intensified at 30 and 40 m. At 50 m, the eddy dissipated, although at 60 and 70 m, a little eddy was present along the upper left-side wall of the drift.
3. The (iii) S1-AVS 3 shows two minor eddies adjacent to the left and right walls at 20 m. At 30 m, an eddy was also present along the left-side wall of the drift. Additionally, a minor eddy formed near the roof at 40, 50, and 60 m. Moreover, an eddy formed below the AVS ducts at 70 m. The (vii) S2-AVS 3 depicts no eddies at 10, 20, and 30 m, respectively. An eddy was present along the right-side wall of the drift at 40 m. Additionally, at 50 m, the eddy migrated to the lower left wall of the drift and diminished in size. Furthermore, the eddy propagated near the roof at 60 and 70 m.
4. The (iv) S1-AVS 4 indicates the presence of two eddies at 20 m, a large eddy beside the left-side wall and a smaller eddy near the right-side wall. At 30 m, the eddy formed adjacent to the left wall of the drift. The eddy expanded in size and persisted at the center of the drift at 30, 40, 50, 60, and 70 m, respectively. The (viii) S2-AVS 4 has no eddies at 10, 20, and 30 m, respectively. An eddy of small size was present along the left-side wall at 40 m. At 50 m, the eddy relocated to a lower center, close to the floor of the drift. Furthermore, at 60 m, the eddy was established between both ducts and near the roof, but at 70 m, the eddy evolved into a larger size and encompassed the middle of the drift.

Overall, the airflow study indicates substantial variations in eddy production and propagation among the AVS designs. AVS 1 and 4 designs typically demonstrate more significant and enduring eddy formations, with eddies originating near walls and extending into the center of the drift. Conversely, AVS 2 and 3 configurations have a reduced number of tiny eddies, characterized by a delayed onset and limited effects, possibly attributable to design changes. These results highlight the impact of AVS design on airflow dynamics and its consequences for drift ventilation efficacy.

### 3.4. Airflow velocity contours

**Fig 9:**
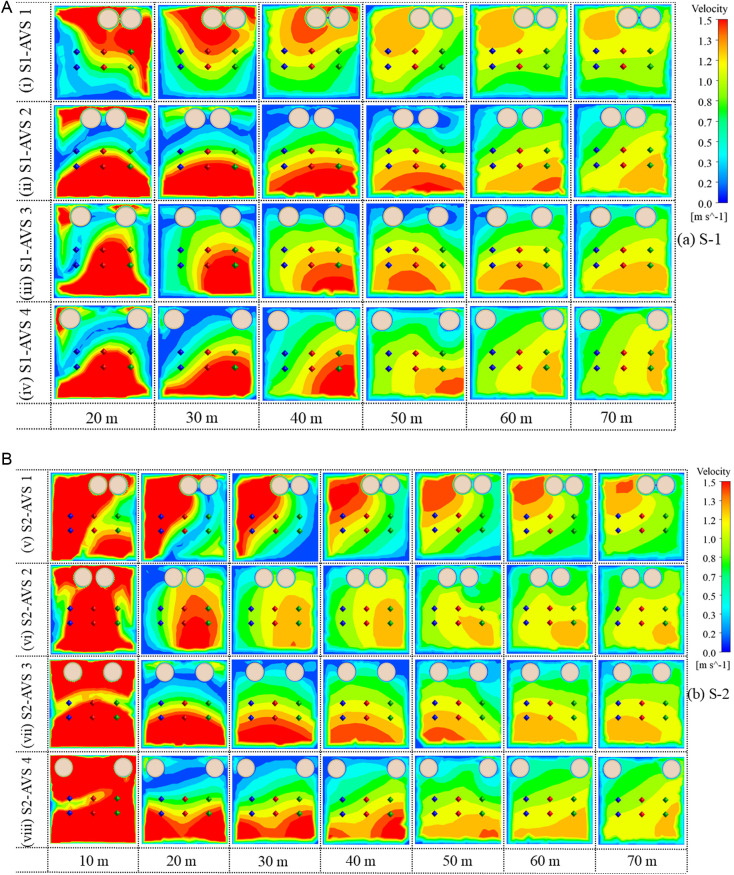
Airflow contour representation on monitoring planes (a) S1 and (b) S2.

The airflow contours illustrate the distribution of airflow concentrations at each monitoring plane in both S1 and S2, as depicted in Fig 9 (a, b). The contours highlight the regions with higher and lower airflow concentrations at each monitoring plane in all four AVS designs. Typically, airflow velocity facilitates the PM dispersion in the underground mine environments.

1. In (i) S1-AVS 1, elevated airflow velocity adjacent to the right-side wall, roof, and surrounding the AVS ducts at 20 m. At 30 m, the elevated airflow velocity was observed near the roof and center of the drift. Moreover, at 40, 50, 60, and 70 m, the elevated airflow velocity was directed towards the right-side wall of the drift. The (v) S2-AVS 1 exhibits the elevated airflow velocity detected on the left-side wall and the bottom right-side of the drift at 10 m. Furthermore, at distances of 20, 30, and 40 m, the elevated airflow velocity was seen adjacent to the right-side wall. Moreover, at 50, 60, and 70 m, elevated airflow velocity was seen at the roof and the upper left wall of the drift.
2. The (ii) S1-AVS 2 exhibits elevated airflow velocity from the center to the floor and toward the roof, resulting in a limited region of low airflow velocity in the middle at 20 m. Furthermore, at 30, 40, and 50 m, elevated airflow velocity was observed from the center to the bottom of the drift. At 60 and 70 m, the elevated airflow velocity transitioned to the lower right-side wall of the drift. The (vi) S2-AVS 2 has a greater airflow velocity, nearly encompassing the entire drift at 10 m. Furthermore, at a distance of 20 m, the elevated airflow velocity was seen from the center to the floor and adjacent to the right-side wall of the drift. Subsequently, a comparable airflow velocity pattern was observed between 30 and 70 m.
3. The (iii) S1-AVS 3 exhibits increased airflow velocity from the center to the floor of the drift at 20 m. Moreover, elevated airflow velocity was observed from the center to the floor and the right-side wall of the drift, spanning 30–70 m. The (vii) S2-AVS 3 exhibits a superior airflow velocity in the entire drift at 10 m. At 20, 30, and 40 m, the elevated airflow velocity was seen from the center to the floor of the drift. At 50, 60, and 70 m, the airflow velocity transitioned to the bottom left side and near the floor of the drift.
4. The (iv) S1-AVS 4 exhibits increased airflow velocity from the center to the floor of the drift at 20 m. Additionally, at 30 m, the airflow velocity was directed toward the left wall of the drift. At 40, 50, 60, and 70 m, the elevated airflow velocity was concentrated from the center towards the left wall of the drift. The (viii) S2-AVS 4 has a higher airflow velocity encompassing the entire drift at 10 m. At 20, 30, and 40 m, the airflow velocity was transmitted to the lower half of the drift, spanning from the left to the right side of the drift. Additionally, at 50, 60, and 70 m, the elevated airflow velocity was observed near the floor, inclined towards the right-side wall of the drift.

The evaluation of airflow velocity across the four AVS designs reveals significant discrepancies in airflow distribution and concentration. AVS designs 2 and 3 typically display specific regions of increased airflow velocity, particularly at the center and near the floor of the drift. While, AVS 1 and 4 have greater airflow near the roof and side-walls of the drift. The impact of the AVS design on airflow dynamics revealed the necessity for optimizing ventilation design and reducing working exposure for miners.

### 3.5. Spatiotemporal distribution characteristics of PM

**Fig 10:**
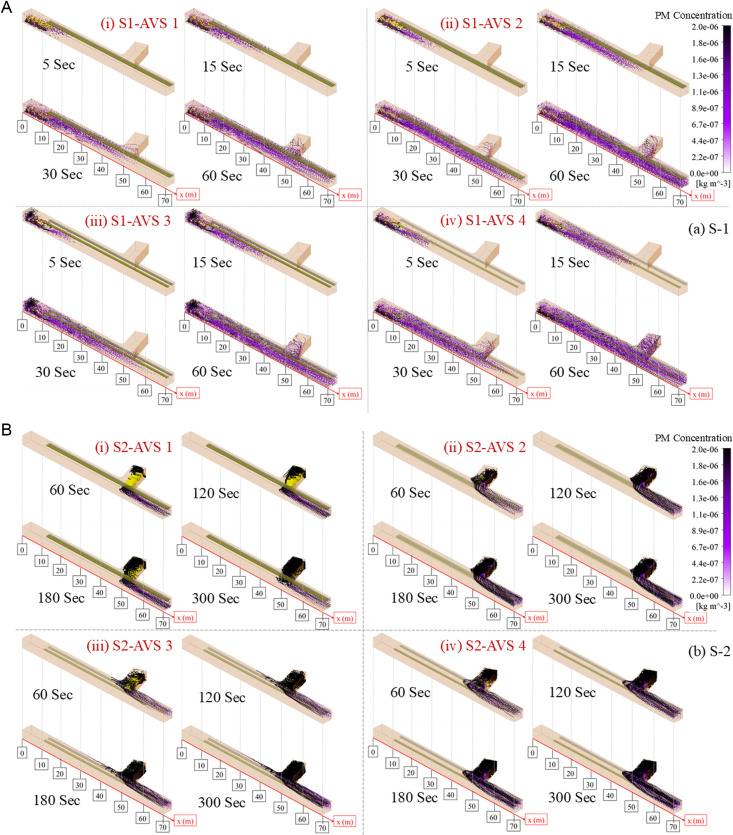
Spatiotemporal PM distribution in (a) S1 and (b) S2.

The spatiotemporal distribution characteristics of PM provide an insight into the distribution of PM in the drift with respect to time. All of the AVS designs in both scenarios, as shown in Fig 10, are evaluated to identify the optimal AVS design for minimizing PM exposure to miners in the drift.

1. In (i) S1-AVS 1 most PM concentrations traveled near the roof and covered 20 m in 5 s. The PM concentration reached over 40 m, while concentrated near the roof in 15 traveled over 50 m in 30 s, and accumulated in the temporary dumpsite. After 60 s, the PM passed over 70 m and exited the drift. In (v) S2-AVS 1 the PM emitted from the DPE tailpipe escaped the drift outlet in 60 s, but the PM generated by DPE unloading the muck moves slowly since the area is not under direct supply of airflow. PM exited the temporary dumpsite in 120 s, whereas DPE tailpipe exhaust did not alter. In 180 s, PM covered most of the temporary dumpsite and accumulated in the drift. In 300 s, the PM from unloading operation mixed with the PM from the DPE tailpipe and exited the drift outlet.
2. In (ii) S1-AVS 2 PM traveled about 30 m in 5 s and most PM concentrations are near the floor. In 15 s, the PM concentration reached 50 m, and while traveling, it stayed concentrated near the floor. In 30 s, the PM accumulated inside the temporary dumpsite and reached the drift outlet. In 60 s, the PM further penetrated the temporary dumpsite. In (vi) S2-AVS 2 both the PM generated by unloading and the PM emitted from the DPE tailpipe exited the drift outlet in 60 s. However, the DPE unloading PM partially covered the temporary dumpsite. In 120 s, the PM at the back of DPE blended with tailpipe exhaust PM. The PM concentration did not change much in 180 and 300 s, and the PM transition from temporary dumpsite to drift exit was smoother.
3. In (iii) S1-AVS 3 most PM concentrations gathered in front of the DPE and traveled less than 30 m in 5 s. In 15 s, the PM concentrations reached 40 m and are concentrated near the floor. In 30 s, the PM concentration reached 60 m but did not accumulate in the temporary dumpsite. In 60 s, the PM gathered deep inside the temporary dumpsite and exited the drift outlet. In (vii) S2-AVS 3 the PM from unloading exited the temporary dumpsite in anti-flow, while the PM from the DPE tailpipe exited the drift outlet in 60 s. At 120 s, the PM concentrated above the DPE and near the roof at the temporary dumpsite and continued anti-flow, while DPE tailpipe exhaust PM transmitted along the right-side wall and roof. In 180 s, DPE unloading PM crossed 30 m in anti-flow direction and nearly covered the temporary dumpsite. In 300 s, the PM concentration did not change significantly, and PM transitioned from temporary dumpsite into two directions simultaneously, towards the working face and towards the drift exit.
4. In (iv) S1-AVS 4 PM traveled about 30 m in 5 s, with higher concentrations near the floor. The PM concentrations reached 40 m in 15 s in the drift and stayed near the floor while traveling. The PM concentration travelled 60 m in the drift in 30 s and began to collect in the temporary dumpsite. The PM penetrated the temporary dumpsite, covered the drift, and departed the drift outlet in 60 s. However, in (viii) S2-AVS 4 the PM collected deep inside the temporary dumpsite, some in the anti-flow direction, and most along the drift’s right-side wall and near the roof in 60 s. In 120, 180, and 300 s, the transition of PM from temporary dumpsite to drift outlet was nearly identical.

The spatiotemporal investigation of PM distribution reveals diverse patterns under different AVS designs in both scenarios. AVS 1 and 4 generally demonstrate reduced PM mobility and increased accumulation at the temporary dumpsite. In contrast, AVS 2 and 3 configurations exhibit enhanced PM dispersion and expedited drift escape, owing to improved airflow patterns and diminished accumulation at the temporary dumpsite. The essential function of AVS design is reducing PM exposure, providing a cleaner and safer working environment for miners.

### 3.6. Analysis of PM contours

**Fig 11:**
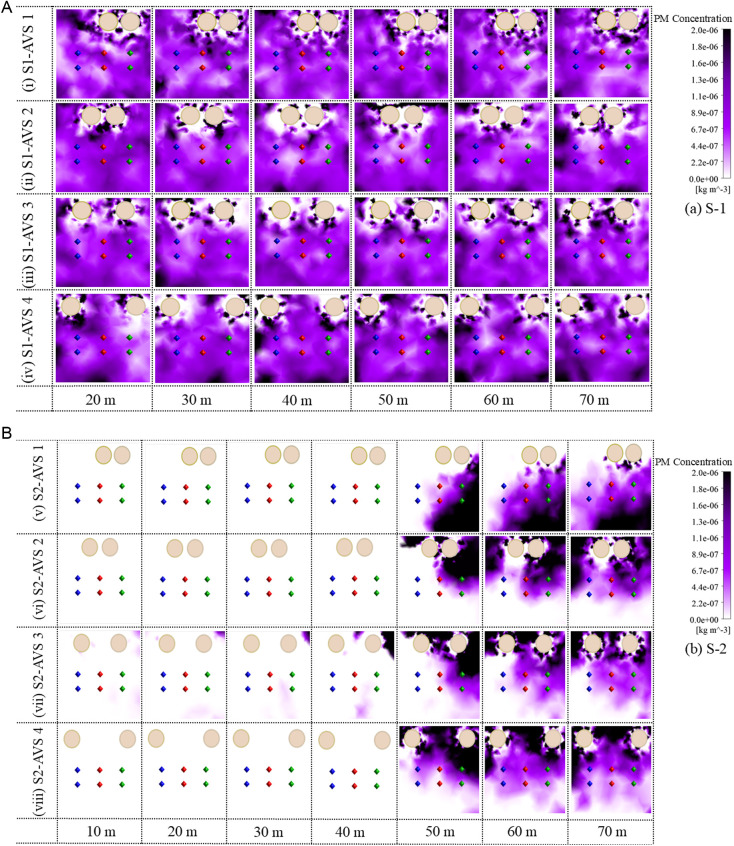
PM concentration on monitoring planes (a) S1 and (b) S2.

The PM contours indicate the distribution characteristics on the monitoring planes in both scenarios and each AVS design, as illustrated in Fig 11 (a, b). These PM contours provide an assessment of regions with the most possibility of PM exposure.

1. In S1-AVS 1, elevated PM concentrations are detected in the upper left quadrant, around the AVS ducts, and adjacent to the right wall and floor within 20–40 m. The PM concentration remains similar around the AVS but changes toward the left wall between 50 and 70 m. The (v) S2-AVS 1 exhibits a lack of PM concentration between 10 and 40 m because the DPE is positioned inside the temporary dumpsite. At 50 m, PM concentration is confined near the lower corner of the right wall. Additionally, at 60–70 m, the PM concentration begins to extend from the right wall towards the left wall along the floor.
2. In (ii) S1-AVS 2 the PM concentration is elevated around the AVS ducts and at the floor at 20 m, with the higher PM concentration persisting near the floor between 30–50 m. The PM concentration persisted at elevated levels along the floor and the left-side wall between 60 and 70 m. In (vi) S2-AVS 2 there is no PM concentration between 10 and 40 m. Nevertheless, the PM concentrations remained concentrated in the upper right corner at 50 m, subsequently transferring from the right side to the left side between 60 and 70 m.
3. In (iii) S1-AVS 3 the PM concentration is elevated around the AVS ducts and adjacent to the left-side wall and floor at 20 m, with the higher PM concentration marinated near the left-side wall and floor between 30–70 m. In S2-AVS 3 has a partial concentration of PM between 10–40 m, owing to a substantial vortex zone inside the airflow field. However, an extensive PM concentration existed in the upper right corner at 50 m, subsequently transferring from the right side to the left side between 60 and 70 m.
4. In (iv) S1-AVS 4 the PM concentration is elevated along the left-side wall and close to the floor at 20 m, and it persists near the floor at 30 m. The PM concentration remained elevated at the left wall and the floor between 40 and 70 m. In (viii) S2-AVS 4 there is an absence of PM concentration between 10 and 40 m. Nevertheless, the PM concentrations remained concentrated near the roof and right-side wall at 50 m, subsequently propagating from the right side to the left side between 60–70 m, encompassing the whole monitoring plane.

The PM contours revealed distinct distribution characteristics under the examined AVS designs and in both scenarios. In S1, the airflow field is more turbulent, so the PM concentration frequently transmits between the monitoring planes. In S2, the position of DPE limited the dispersion of the PM on the monitoring planes., AVS 2 and 3 designs show more promising control over the dispersion of the PM in the drift and reduce the potential PM exposure to miners.

### 3.7. Comparative analysis of PM concentration under AVS 1–4

**Fig 12:**
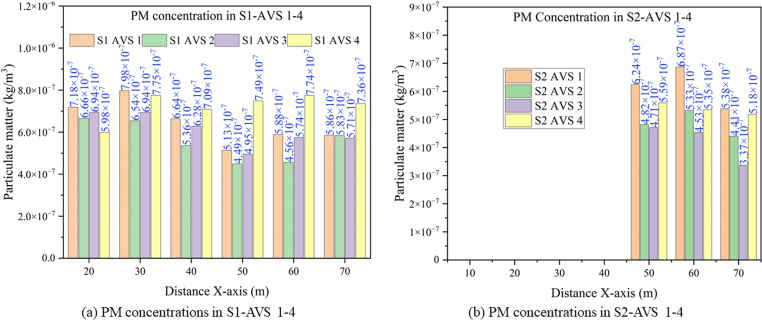
Comparison of PM concentrations under AVS 1-4 in (a) S1 and (b) S2.

The graphs provide the PM concentration under AVS 1–4 and in both scenarios. Notably, an average of all six monitoring points is considered as an average PM concentration at each of the monitoring planes as shown in Fig 12 (a) S1 and (b) S2. The turbulent airflow in the mine drift resulted in frequent fluctuations of PM concentrations along the x-axis direction.

1. In S1-AVS 1, the PM concentration varied, reaching a maximum of 7.98x10^-7^ kg/m^3^ at 30 m to a minimum of 5.86x10^-7^ kg/m^3^ 70 m. For S1-AVS 2, the PM concentration peaked at 6.66x10^-7^ kg/m^3^ at 20 m and dropped to a minimum of 4.49x10^-7^ kg/m^3^ at 60 m. In S1-AVS 3, the maximum and minimum PM concentrations of 6.94x10^-7^ and 4.95x10^-7^ kg/m^3^ were observed at 20 and 50 m, respectively. In S1-AVS 4, the maximum PM concentration attained was 7.75x10^-7^ at 30 m, while the minimum concentration of 5.98x10^-7^ kg/m^3^ occurred at 20 m.
2. Conversely, in S2-AVS 1, the PM concentrations ranged from a maximum of 6.87x10^-7^ kg/m^3^ at 60 m to minimum of 5.38 x10^-7^ kg/m^3^ at 70 m. For S2-AVS 2, the maximum PM concentration was 5.33x10^-7^ kg/m^3^ at 60 m, while the minimum was 4.41x10^-7^ kg/m^3^ at 70 m. In S2-AVS 3, the maximum and minimum PM concentrations of 4.71x10^-7^ and 3.37x10^-7^ kg/m^3^ were observed at 60 and 70 m, respectively. Lastly, in S2-AVS 4, the highest PM concentration of 5.59x10^-7^ kg/m^3^ was observed at 50 m, while the lowest value of 5.18x10^-7^ kg/m^3^ was recorded at 70 m.

The results indicated that the comparison of AVS 1 with AVS 2, AVS 3, and AVS 4 showed a significant change in the PM dispersion. In S1, the PM dispersion improved by 15.66% and 7.83% in AVS 2 and AVS 3, respectively, but decreased by 9.44% in AVS 4. In S2, the PM dispersion improved by 27%, 46%, and 14.6%, in AVS 2, AVS 3, and AVS 4, respectively. The results also demonstrate the variability of PM concentration based on the type of AVS employed to control the PM concentration. Notably, the placement of AVS duct outlets affects the dispersion and regulation of PM in underground environments.

## 4. Field monitoring

### 4.1. Data monitoring points and planes

To validate the precision of the numerical simulation results, we conducted field measurements of airflow velocity and PM diffusion in the mine drift. The field measurement outcomes were compared with the modeling results to ascertain precision and variance. In both scenarios, six monitoring points were chosen by considering the breathing zone for miners at a height of 1.5 m and the breathing zone for LHD operators at a height of 2.2 m from the ground. The xyz coordinates of the six monitoring points are as follows: 1(x, 1.5 m, 3.4 m), 2(x, 1.5 m, 2.2 m), 3(x, 1.5 m, 1 m), 4(x, 2.2 m, 1 m), 5(x, 2.2 m, 2.2 m), and 6(x, 2.2 m, 3.4 m).

**Fig 13:**
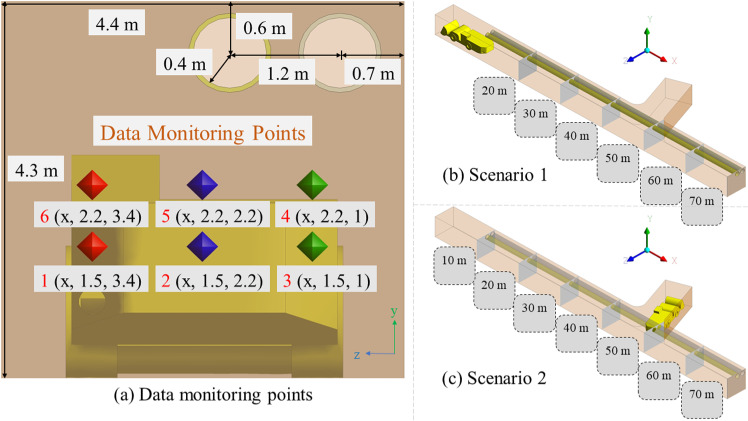
(a) Data monitoring points and planes in (b) S1 and (c) S2.

In S1, six monitoring planes were positioned at distances of 20 m, 30 m, 40 m, 50 m, 60 m, and 70 m from the working face, whereas seven monitoring planes were positioned at distances of 10 m, 20 m, 30 m, 40 m, 50 m, 60 m, and 70 m from the working face. The coordinates of the monitoring sites are depicted in Fig 13 (a), while the arrangement of the monitoring planes for S1 is illustrated in Fig 13 (b) and for S2 in Fig 13 (c).

### 4.2. Comparison of field monitoring and simulation results

**Fig 14:**
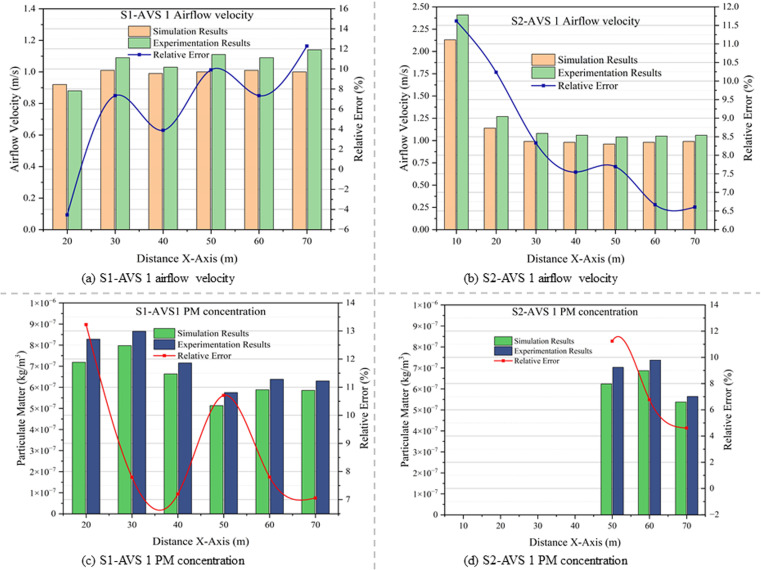
Comparison of experimental results and simulation results of airflow velocity (a) S1-AVS 1, (b) S2-AVS 2, and PM concentration (c) S1-AVS 1, (d) S2-AVS 2.

The data collected from experimentation at each monitoring point and plane was evaluated to determine the average airflow velocity and PM. The simulation results closely align with the experimental results in both scenarios, indicating the authenticity of the simulation model. Mainly, the relative error stayed below 10%, and rarely exceeded 10%. This variance can be ascribed to dynamic operating conditions, wall roughness, the presence of individuals during data collection, and the immobile posture of a human hand during field experiments. In engineering applications, a 20% relative error between simulation and experimental results is typically deemed acceptable [48,49]. Fig 14 (a, b) displays a comparison study of experimental and simulated airflow velocity results, together with the relevant errors in both scenarios. The experimental and simulated PM concentrations, along with the relevant errors in both scenarios, are depicted in Fig 14 (c, d).

## 5. Conclusion

This research employs Ansys-Fluent numerical simulation to assess the distribution and migration of the airflow field and PM in two distinct scenarios across four AVS designs. The simulation results were validated against field experimentation outcomes. The impact of various AVS designs on PM dispersion and dilution was examined to offer theoretical insights for determining the optimal AVS design that can successfully reduce mine worker exposure and enhance the underground mining environment. The subsequent findings are derived:

The entrainment effect of AVS, restricted space of drift, and the location of the DPE result in airflow streamlines being distributed into backflow and vortex regions. These regions were crucial in the dispersion and dilution of PM concentration, contingent upon their size and airflow velocity. The dimensions of each regions exhibited minor variations in both scenarios and across all AVS designs.

This study used two common scenarios in underground mines that highlight the health hazards encountered by miners. First, the DPE operator faces a possibly higher exposure risk due to a significantly reduced PM diffusion rate within the temporary dumpsite (seen in S2). Second, the DPE operator faces serious risks of elevated PM exposure due to the formation of Backflow and Vortex zones, as seen in S1. These common scenarios are the main reasons for obligating the improvement of the AVS to mitigate PM exposure among underground miners.

The findings underscore the efficacy of AVS 2 and AVS 3 in reducing PM exposure, placing them as the optimal designs for improved air quality management. In both S1 and S2, the numerical simulation analysis indicated that on average PM dispersion was increased under AVS 2 and AVS3. In S1, 15.66% and 7.83% PM dispersion were attained under AVS 2 and AVS 3, respectively, and in S2, dispersion increased by 27% and 46%, under AVS 2 and AVS 3, respectively. Additionally, the PM contours and spatiotemporal analysis indicated that these designs enabled a distinctly quicker transfer of PM from its source to the drift exit in comparison to AVS 1 and AVS 4.

To further reduce PM emissions, significant idle durations should be curtailed, or engines should be turned off if lengthy idling is anticipated. Although dynamic operations of DPE are essential to the mining transportation system, health concerns related to PM generation can be substantially reduced by optimizing the work schedule and the optimized AVS design.

### 5.1. Limitations of the study

Although the extensive outcomes of the present study, it is crucial to acknowledge certain limitations for future investigations. This study examined airflow velocity and PM concentrations in the studied AVS designs; however, field experimentation was conducted solely under AVS 1 in both scenarios. Furthermore, the assessment of the exposure length of the DPE operator to the areas with elevated levels of PM was not considered. Furthermore, a link between the total ore transport rate and PM concentration can yield substantial insight into miners’ potential exposure. Therefore, future studies must concentrate on these traits to provide substantial insights on reducing miners’ exposure to elevated amounts of PM.
